# Time series evaluation of condemnation at poultry slaughterhouses enable to export in Southeastern Brazil (2009–2019): a tool for optimizing resources in the poultry production chain

**DOI:** 10.1186/s12917-022-03521-z

**Published:** 2022-12-08

**Authors:** Maria Carolina Hortêncio, Letícia Roberta Martins Costa, Maria Victória Pereira de Souza, Weslley Domenicci de Freitas, Belchiolina Beatriz Fonseca, Marcelo José Barbosa Silva, Marcus Vinícius Coutinho Cossi

**Affiliations:** 1 Federal Inspection Service, Ministry of Agriculture, Livestock and Supply (MAPA), Uberlândia, Minas Gerais Brazil; 2 Federal University of Uberlândia (UFU), Campus Umuarama, Uberlândia, Minas Gerais 38405-240 Brazil

**Keywords:** Turkey, Broiler, Inspection, Food safety

## Abstract

**Background:**

Even with the technological advances in management, health and genetics applied to poultry farming worldwide, there is still a high rate of carcasses condemnation at slaughterhouses, which result in losses for the poultry production chain. Thus, this work aimed to evaluate the condemnation occurrence index (COI) and adjusted seasonal index (ASI) of poultry (turkey, griller, and heavy chicken) between 2009 and 2019, in a slaughterhouse enable to export in southeastern Brazil. Data were obtained from official spreadsheets from the Brazilian Federal Inspection Service (FIS) and used to calculate the COI, correlation analysis between the main causes of condemnation, and ASI assessments throughout the year.

**Results:**

Seven percent (55,594,318) of the poultry carcasses were condemned (partial or total), and the most frequent causes, contamination, and contusion/traumatic injury, amounted to 63.5% of the total condemnation. There was a trend of increasing condemnation throughout the time series evaluated, with COI varying between 45,282–149,809 condemnations per 1,000,000 poultry slaughtered. Considering the ASI, it was identified that for ascitic syndrome, July has a higher index value (1.63) than the months between January–June (*P* < 0.05).

**Conclusions:**

The main causes of condemnation were contamination and contusion/traumatic injury, both technological causes. ASI showed that in July there is a greater carcasses condemnation due to ascitic syndrome than in the months between January and June. The variations observed in the ASIs can provide subsidies for preventive measures and optimization of human and financial resources, generating positive impacts on food safety, productivity, and profitability of the sector.

## Background

In Brazil, all slaughterhouses must be registered with an official inspection service (municipal, state, or federal), and exporters must be registered under the Federal Inspection Service (FIS). One of the functions of the FIS is to inspect and supervise the production process at the slaughterhouse, to guarantee the hygienic-sanitary quality of the end product. As part of this activity, official veterinarian inspectors should carry out ante-mortem and post-mortem inspections of animals [1].

Among their responsibilities, the official veterinarian is responsible for inputting the daily condemnation data into the Management Information System of the Federal Inspection Service (MISFIS) [1, 2]. Considering the importance of Brazil as a producer and supplier of animal protein to the world, studies have used the daily condemnation data to analyze and propose improvements that contribute to the sector [3–5].

Condemnation data from Brazil and other leading countries in poultry production show variations in frequency and causes, highlighting the need for constant monitoring of all production chain process [6–8]. Understanding the reality of each region and how the variables interfere in the condemnation rates is how personalized interventions can guarantee greater profitability for the production chain [7, 9–11].

Among the possible causes of condemnation there are technological and non-technological causes. Non-technological causes are related to the presence of a pathogen, poor hygienic conditions in production, and climatic variations that can result in the development of aerosacculitis and arthritis, for example [11, 12]. On the other hand, technological condemnations are the result of failures in pre-slaughter and slaughter management stages, such as catching, transport, hanging, and scalding the poultry. Each cause of condemnation, technological or non-technological, is evaluated in terms of its extension in the carcass, and impact on the food quality and safety, resulting in the decision for its partial or total condemnation [1]. These are common causes of condemnation, reinforcing the need for data analysis that contributes to the development of effective training programs applied to the production and inspection stages [13–15].

Thus, the present study aimed to evaluate the historical behavior of the condemnation occurrence index (COI) and adjusted seasonal index (ASI) of poultry in a slaughterhouse in southeastern Brazil from 2009 to 2019, to contribute information that helps food safety (and food security.

## Results

**Fig. 1:**
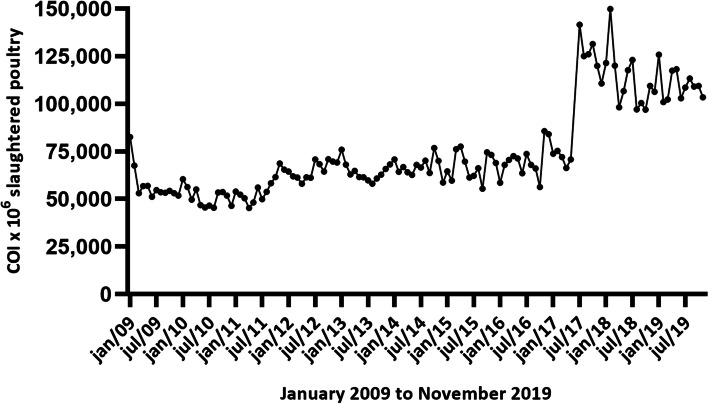
Condemnation Occurrence Index (COI) of poultry slaughtered in an export slaughterhouse, located in Brazil (2009–2019). *June 2017 and December 2019 were removed from the analyzes due to inconsistencies in their data

Between January 2009 and November 2019, 785,068,807 poultry were slaughtered, 95% of which were broilers and 5% were turkeys. Of this total number of animals slaughtered, 7.08% (55,594,318) suffered some type of condemnation (partial or total). Based on these data, the rate of the COI was calculated, and the results are shown in Fig. 1. During the period evaluated, the COI varied from 45,282 to 149,809 per million poultry slaughtered, with an upward trend observed during the analyzed historical period.

It was found that, between 2009 and 2016, an average of 5,023,045 turkeys were slaughtered per year. In 2017, the total number of turkeys slaughtered was only 1,789,429, a reduction of 64.38% compared to the average number of turkeys slaughtered in previous years. In 2018, these animals were slaughtered only in January and February, making a total of 258,773 turkeys. In addition, griller chickens were slaughtered until June 2017, and from July 2017, the slaughterhouse began to slaughter heavy chickens instead of griller chickens. These changes in the profile of slaughtered animals coincide with the increase in COI values observed in the figure from July 2017 (Fig. 1).

The condemned poultry carcasses were then evaluated in terms of their causes recorded in the official spreadsheets (Table 1). Considering only the four main causes in this evaluated slaughterhouse (contamination, contusion/traumatic injury, dermatitis, and aerosacculitis), there were 45,044,707 condemnation records (total or partial), which represented 84.93% of the total number of finds in the period. It should be noted that the first two (contamination, contusion/traumatic injury) were considered technological failures, which together represented 63.51% of the total number of condemnations observed in the period. The two subsequent causes (dermatitis and aerosacculitis) added up to 11,360,680 (21.42%) of condemnations and were not considered technological failures.

**Table 1:** Frequency of poultry condemnations (partial + total) slaughtered in an export slaughterhouse, located in southeastern Brazil (2009–2019)

Causes	Technological failure	Number	%
Contamination	Yes	19,714,313	37.17
Contusion / Traumatic Injury	Yes	13,969,714	26.34
Dermatosis	No	6,305,341	11.89
Aerosacculitis	No	5,055,339	9.53
Arthritis	No	2,120,165	4.00
Septicemia	No	1,712,767	3.23
Disgusting aspects	No	1,665,298	3.14
Excessive scalding	Yes	1,133,914	2.14
Cellulitis	No	889,035	1.68
Others *		478,617	0.90
**Total**		55,594,318	100.00

*Bloody meat, Ascites, Cachexia, Delayed evisceration, contagious epithelioma

Considering the interruption of turkey and griller chickens’ slaughter, the beginning of the heavy chickens’ slaughter, and the increase in condemnations observed in Fig. 1, a comparison was made between Groups A and B (Table 2). It was observed that only in the case of aerosacculitis, the non-slaughter of turkey and griller chickens resulted in a reduction in the average condemnation per total number of poultry slaughtered (*P* < 0.001). Corroborating this finding, the average COI per aerosacculitis, between 2009 and 2016, was 7491.73 per million birds slaughtered, in 2017 (with a 64.38% reduction in turkey slaughter and interruption of griller chicken slaughter in June) the index was 6237.91, and between 2018 and 2019 (turkey slaughter only in January and February 2018) the index was only 168.2.

**Table 2:** Frequencies of carcass condemnation in months with turkey and chicken slaughter and months with chicken only slaughter in a slaughterhouse registered with the Federal Inspection Service, located in southeastern Brazil (2009–2019)

Evaluated variables	Group^a^	n^b^	Mean	SD	SE	***P*** value	Rank-Biserial Correlation
Total condemnation / Total slaughter	A	110	6.925	2.716	0.259	< 0,001	−0.848
	B	21	10.892	0.872	0.190		
Contamination/Total Slaughter	A	110	2.322	0.639	0.061	< 0,001	−0.935
	B	21	4.484	0.408	0.089		
Aerosacculitis /Total Slaughter	A	110	0.787	0.490	0.047	< 0,001	1.000
	B	21	0.001	0.005	0.001		
Contusion and Traumatic Injury / Total Slaughter	A	110	1.658	0.670	0.064	< 0,001	−0.855
	B	21	3.250	0.292	0.064		
Dermatosis/Total slaughter	A	110	0.798	0.375	0.036	< 0,001	−0.681
	B	21	1.065	0.147	0.032		

^a^A: turkey and griller chicken slaughter; B: slaughter of heavy chicken

^b^number of months in which turkey and griller chicken were slaughtered (A), or only heavy chicken were slaughtered (B)

For the findings of contamination, contusion, and dermatitis, the result was the opposite (Table 2). Group A had the lowest average condemnations for these causes, with values 2.16, 1.59, and 0.27%, respectively lower (*P* < 0.001). This demonstrates a greater detection of these causes of condemnation in heavy chickens when compared to the findings in the slaughter of turkeys and griller chickens. The analysis of total condemnation by the total slaughtered animals also showed the higher proportional occurrence of condemnations in heavy chickens (Group B), since there was a 3.97% increase in the average number of condemnations when compared with Group A (*P* < 0.001).

**Fig. 2:**
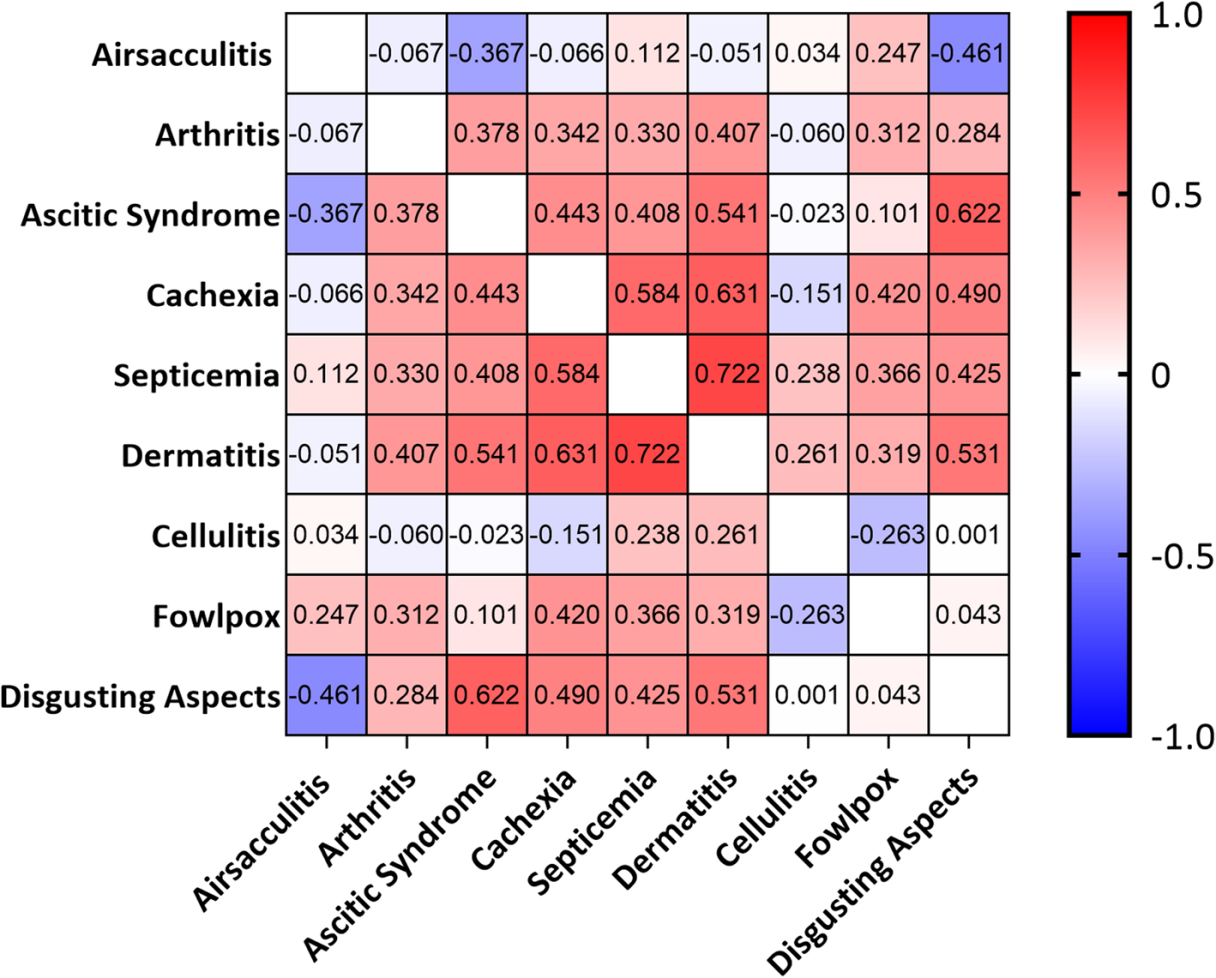
Correlation between causes of poultry condemnation in a export slaughterhouse, located in Brazil (2009–2019)

Then, the correlation presented between the main diseases studied in this work was evaluated (Fig. 2). A high positive correlation was identified between septicemia and dermatitis (0.722), moderate correlation between ascitic syndrome and dermatitis (0.541), ascitic syndrome and disgusting aspects (0.622), cachexia and septicemia (0.584), cachexia and dermatitis (0.631), and between dermatitis and disgusting aspects (0.531).

**Fig. 3:**
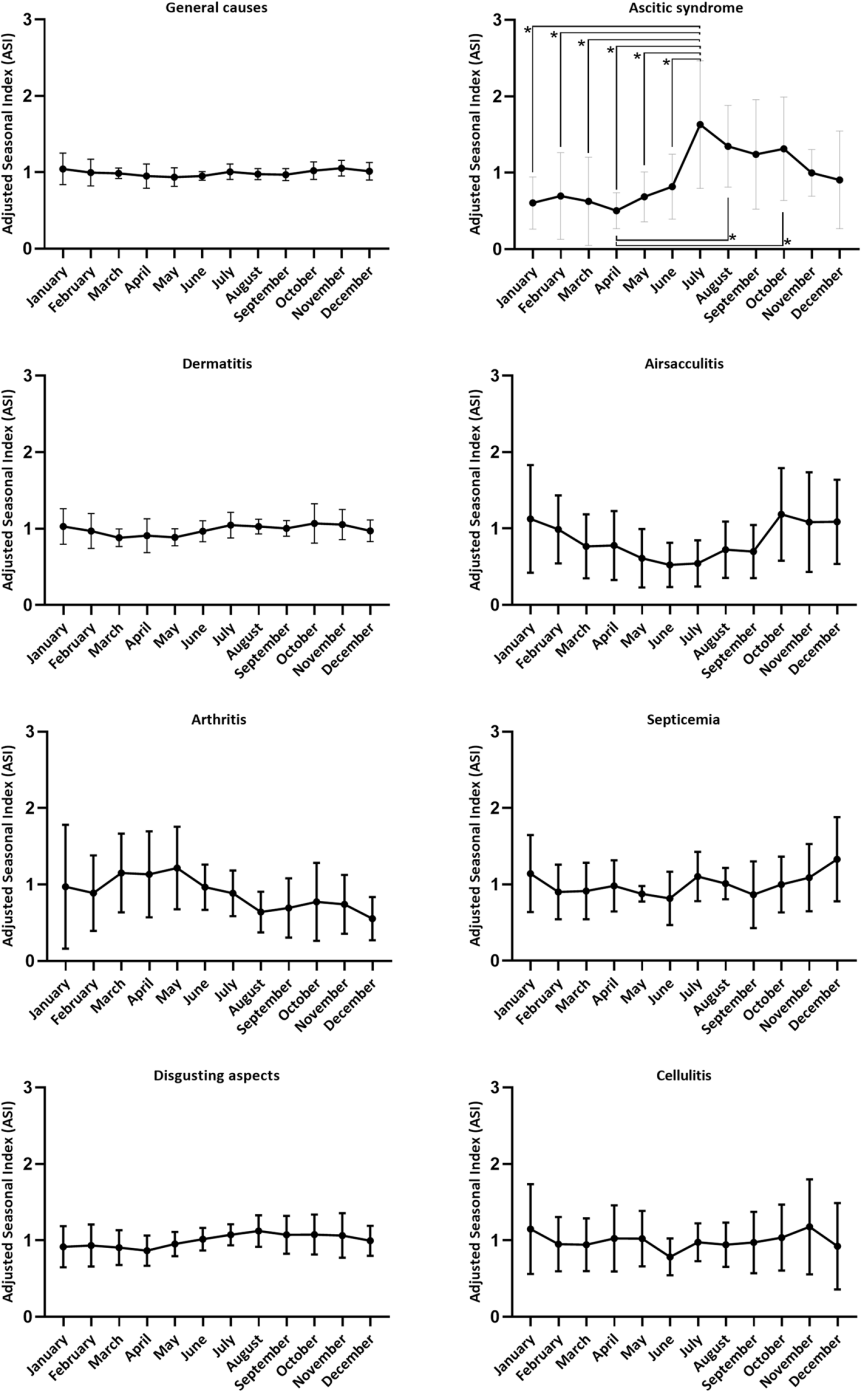
Adjusted Seasonal Index (ASI) condemnation in export poultry slaughterhouse, located in Brazil (2009–2019)

To assess the behavior of condemnations throughout the year, the ASI was then evaluated (Fig. 3). Despite the variations in the ASI throughout the year, only for ascitic syndrome were these differences significant (*P* < 0.05). For ascitic syndrome, there was a variation from 1.63 to 0.5 in the ASI, indicating, respectively, an increase of 63% and a reduction of 50% in condemnations, in relation to the average value expected for the year. The highest ASIs were identified from July to October, however, a statistical difference was only observed between July and the months from January to June (*P* < 0.05).

**Fig. 4:**
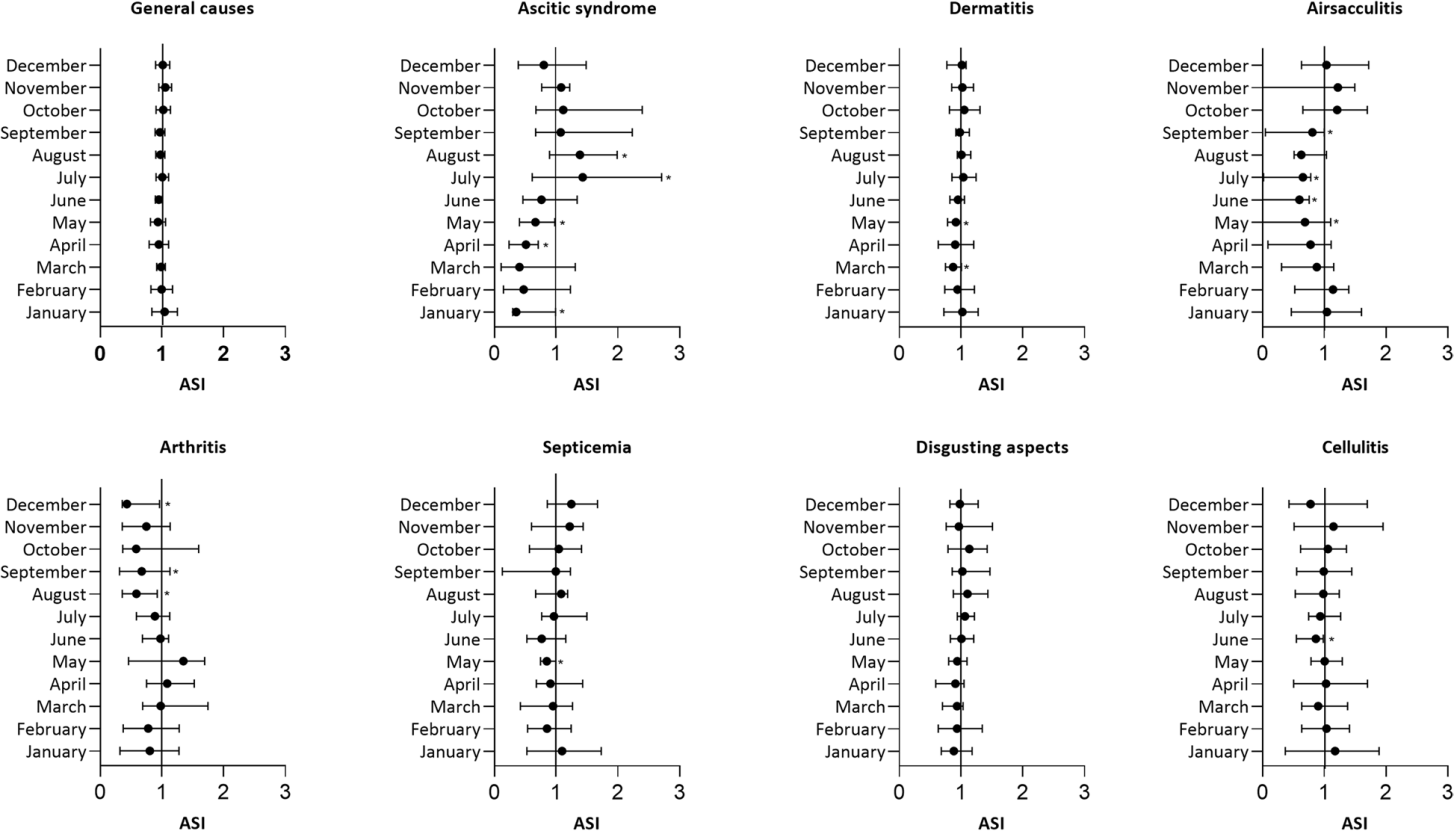
Adjusted Seasonal Index (ASI) and historical average of condemnations in export Brazilian poultry slaughterhouse

Finally, the variation that each month had in relation to the average condemnations expected for the year (1 = 100%) was analyzed (Fig. 4). Considering all the causes of condemnation, June was the only month in which there was a significant variation, with a 5% reduction in condemnation rate. For all causes of condemnation evaluated, there was at least 1 month with a condemnation rate different from the year average (*P* < 0.05). Aerosacculitis and ascitic syndrome were the causes of condemnations with the highest number of months showing significant variations (5 and 4 months, respectively). For aerosacculitis, a significant reduction was observed from May to September, with values ranging from 28 to 48% (*P* < 0.05). For ascitic syndrome, July had a 48% increase in condemnation, while January, April, and May had a reduction ranging from 31 to 39% (*P* < 0.05).

## Discussion

The tendency of increase observed in the present study (Fig. 1) showed that the COI of poultry carcasses is a useful tool in the identification of historical variations of condemnation. These trends reflect technologies used, sanitary conditions of the batch, and employee training policies [3–5]. Therefore, the proper recording and analysis of the occurrences of condemnation in a slaughterhouse are essential for the planning of preventive measures, aiming at economic gains for the meat supply chain [10, 11]. In addition, they may show behavioral changes in condemnation rates and indicate the need for specific investigations, such as the changes observed in COIs from 2017 onwards (Fig. 1).

Concurrent with the increase in slaughterhouse COI, there was a reduction in turkey slaughter, with a subsequent discontinuation and replacement of broiler slaughter by heavy chicken slaughter. Evaluating Groups A and B, it was evidenced that there was a higher rate of condemnation in the period in which only heavy chickens were slaughtered (Table 2). The higher age of these animals at slaughter, compared to griller chickens, and the high stocking rate of the sheds were factors directly linked to the increase in the occurrence of condemnation [16]. Turkeys showed little impact on this COI variation, as they represented only 5% of the total number of animals slaughtered in the period evaluated. Furthermore, changes in Brazilian legislation in 2017 and 2019 impacted carcass disposal standards and increased the number of inspection agents in the slaughter process. Added to this, the greater incentive to train teams, from 2018 onwards, with a focus on ante and post-mortem inspection, may also have contributed to this finding [1, 17, 18].

In agreement with other studies our findings showed that condemnation rates can vary from 0.75 to 13.16%, so the result observed in the present work has an intermediate value, with 7% of condemnations between 2009 and 2019 [4, 6–8, 19]. However, the direct comparison between condemnation rates from different countries must be done with caution since there are variations in the legislation and the criteria for the destination of the carcass. In addition, characteristics of climate, altitude, production systems, and even social differences may contribute to the observed variations [9, 10, 20–22].

The relative contribution of the causes of condemnation of poultry carcasses highlights the challenges that need to be faced by the poultry industry (Table 1). These challenges impact differently in each region and country as they are influenced by management failures, the slaughter process, or poultry flock health [6, 10, 22, 23]. The latter, as it involves etiological agents, tend to be influenced by management and also by geographic and climatic characteristics of each region and season of the year [5, 7, 11].

In the present study, the four main causes of condemnation were: (i) contamination, (ii) contusion/traumatic injury, (iii) dermatitis and (iv) aersacculitis (Table 1)”. Similar results were found in other studies, with erosacculitis being the only difference observed, since for other studies this was not among the main causes of condemnation [4, 7, 8]. This difference can be explained by the slaughter of broilers and turkeys in the evaluated slaughterhouse, the latter being responsible for contributing with the highest number of condemnations for aerosacculitis. The higher occurrence of aerosacculitis in turkeys may be related to the greater age at slaughter, which implies a longer exposure to variables relevant to the development of the disease: handling failures, poor air quality, high concentrations of dust and ammonia, and the presence of pathogenic microorganisms such as *Escherichia coli* and *Mycoplasma* spp. [4, 10, 24].

The high frequency of carcass contamination (37.12%) must be analyzed and prevented by slaughterhouses. According to Brazilian legislation, condemnation by contamination can occur in poultry due to extravasation of gastrointestinal content, bile or any other type of macroscopic contamination identified in the slaughter line [1]. Among the possible causes of contamination, the extravasation of gastrointestinal contents deserves special attention as it is one of the main sources of foodborne pathogens such as *Salmonella* spp., *E. coli*, and *Campylobacter jejuni* [20, 23, 25]. Carcass contamination occurs largely due to difficulties in adjusting equipment during the slaughter of uneven batches, or fasting periods in disagreement with the recommendations [3, 7].

The second main cause of condemnation (injury/traumatic injury), as well as contamination, is also considered a technological failure and is usually related to problems during the catching, transport, or hanging of poultry [22, 26, 27]. Therefore, the integration of the production chain (producers and slaughterhouse) and training focused on good management practices and animal welfare are essential for reducing condemnation [3, 4, 28].

These condemnations resulting from technological failures are part of the daily routine of poultry slaughterhouses. In this way, continuous actions need to be incorporated into the establishments’ practices, such as training and preventive maintenance of equipment, to reduce these problems [3, 29, 30]. It should be noted, however, that specific studies must be adopted in each slaughterhouse to identify the real cause of the problem among all the variables that may be involved.

Dermatitis was identified as the third leading cause of condemnation, being the first when considering those involving pathogenic agents. Dermatitis is the term used by the Brazilian Federal Inspection Service to record skin diseases, with the exception of cellulitis [31]. Its occurrence is related to high stocking rates, bird weight, temperature stress, and litter quality [7, 12].

High and moderate positive correlations between some causes of condemnation were identified in the present study (Fig. 2). Septicemia has already been associated with several bacteria such as *Escherichia coli*, capable of reaching the bloodstream during a case of aerosacculitis or through the intestinal wall, and *Staphylococcus aureus*, which are commonly associated with skin lesions (dermatoses and cellulitis) [3, 10]. In cases of cachexia and disgusting aspects, there are several possible causes associated with this finding, such as malnutrition, which is unlikely in industrial production, management problems, environment, or secondary causes of diseases or pathologies [23, 32]. Therefore, by evaluating the diseases’ predisposing factors and the correlation results, we can understand that actions that improve sanitary and management conditions will bring benefits in the simultaneous reduction of several condemnation causes [4, 12].

The relationship between causes of condemnation and annual variations in rainfall, humidity, and temperature have already been studied by other authors [12, 33]. In the present study, the only cause of condemnation for non-technological failures with an ASI difference was ascitic syndrome, with the highest values identified in the months of July to October (*P* < 0.05) (Fig. 3). A similar result was found by Souza et al. [5] who, analyzing the states with the highest poultry production in Brazil, identified the highest ASIs from July to September. These variations in ASI values can be explained by the fact that ascitic syndrome is a metabolic syndrome, which is caused by interactions between diet, genetic, and environmental factors (altitude and temperature) [34, 35]. Corroborating this information, July to September correspond to the coldest period of the year in the southeastern region of Brazil [5]. Thus, the findings of this work can help in the planning of adequate management in relation to the temperature and ventilation of the sheds in cold climates, reducing the incidence of this syndrome.

Regarding the evaluation of reductions and increases in the ASIs for condemnations not associated with technological failures (Fig. 4), we can use the results obtained as a way of predicting future condemnation in a stable population [33, 36]. This forecast on the occurrence of condemnation is fundamental in determining preventive management procedures and directing the training of inspection and quality control teams and technicians working in the field [4]. This training can prioritize causes of condemnation that are critical in a certain period of the year, instead of causes in which there is a significant reduction in the occurrence. This rational use of financial and human resources can contribute to obtaining more expressive results in the reduction of diseases in the field and in the ease of their identification during the slaughter process [15, 37].

## Conclusion

The main causes of condemnation of poultry identified in this study were contamination and contusion/traumatic injury, both technological causes. There was also a high positive correlation between septicemia and dermatitis. For ascitic syndrome, the ASI of July was higher than the months from January to June. In addition, the variations observed in the ASIs in relation to the average expected for the year can support the best allocation of resources aimed at improving food safety and food security guaranteed by the poultry production chain.

## Methods

### Data collection and calculation of indices and frequencies

A cross-sectional study was carried out using data available on the official website of the Ministry of Agriculture, Livestock and Supply, on the amount of slaughter and condemnation of poultry carcasses between January 2009 and November 2019. The data were from a slaughterhouse located in the southeastern region of Brazil and authorized to export. This slaughterhouse is part of a complete integration structure, called Vertical Integration. Monthly data on slaughtered animals, causes of condemnation (total + partial), and species slaughtered (griller chicken, heavy chicken, and turkey) were obtained. The “griller” chicken comes from industrial strains, selected for better zootechnical performance, slaughtered at up to 35 days of age [38]. The database used for this study does not allow differentiation between total and partial condemnation and, therefore, condemnation was considered as the sum of both. All collected data went through individual verification to identify and exclude inconsistent information.

In these official data consulted, it was possible to identify the months in which turkey, griller chicken, and heavy chicken were or were not slaughtered, however, with the slaughter of both categories of chicken, or species, it was not possible to differentiate the origin of the condemnation (turkey, griller, or heavy chicken). Thus, the months were classified into Groups A (when there was the slaughter of griller chicken and turkey or heavy chicken and turkey) and B (when only heavy chicken was slaughtered). No month was linked with slaughtering only turkey or griller chicken.

The number of condemnations in relation to the number of animals slaughtered was evaluated by the COI, defined as the ratio between the monthly number of condemnations (total + partial) and the total number of animals slaughtered in that period [33]. The resulting index was multiplied by 10^6^ (occurrence of condemnation for every one million poultry slaughtered) in order to facilitate its interpretation. To assess the relative contribution of each cause of condemnation, the frequency of each cause among the total number of condemnations in the period evaluated was also calculated. The causes of condemnation were classified according to their association or not with technological failures. Those that were associated with failures during the harvesting, transport, or slaughtering process were classified as technological failures. The other causes of condemnation, associated with the presence of a pathogenic agent, precarious hygienic conditions in production, or climatic variations, were classified as non-technological failures [11, 12, 39].

The ASI was then calculated for the total condemnation values [39] and also for main causes classified as not associated with technological failures (ascitic syndrome, dermatitis, aerosacculitis, arthritis, septicemia, disgusting aspects, and cellulitis) [33]. First, values of monthly COI were used in the numerator and the annual COI mean in the denominator to identify the specific contribution of each month in the condemnation rates of the year (JAN2009 = COI January 2009/COI 2009 average; FEB2009 = COI February 2009/COI 2009 average … NOV2019 = COI November 2019/COI 2019 average). Later, the ASI was obtained by calculating the average for each month using the 11-year historical series ((JAN2009 + JAN2011 + ... JAN2019)/11; ... (DEC2010 + DEC2011 + ... DEC2018)/10) (data referring to December 2019 were excluded due to inconsistency). Through calculation of the ASI, it was possible to minimize random variations that may have occurred in condemnation occurrence data.

Causes not associated with technological failures were selected for calculating the ASI, considering that the possible participation of an etiological agent in association with environmental factors could give these causes a cyclical behavior throughout the year. In addition, cachexia and contagious epithelioma were not included in the ASI analysis due to their low occurrence observed in the data.

### Data analysis

All the results obtained were on spreadsheets and descriptively evaluated regarding the frequency of occurrence. To compare the difference between Groups A and B, the total occurrence of condemnation and the main causes of condemnation were transformed into categorical variables (0 and 1) and compared by t test (*P* < 0.05). The evaluation of the effect size resulting from the exclusive slaughter of heavy broilers (Group B) was calculated by the point-biserial correlation coefficient. The correlation between causes of condemnation not associated with technological failures was evaluated by Spearman’s rho correlation coefficient heatmap (*P* < 0.05). The strength or degree of correlation was evaluated according to Mukaka [40]. The ASI results were finally compared in two ways. First, the ASI values obtained in each month were compared by the post hoc ANOVA test (Tukey). Then, the monthly ASI values were compared with the expected value for the year using the t test with the hypothetical value set at one (100% of the expected condemnation).

## Data Availability

The dataset analyzed during the current study is available from the corresponding author on reasonable request.

## References

[1] BRASIL. Ministério da Agricultura, Pecuária e Abastecimento. DECRETO N^o^ 9.013 de 29 de março de 2017. Dispõe sobre o regulamento da inspeção industrial e sanitária de produtos de origem animal 2017.

[2] BRASIL. Ministério da Agricultura, Pecuária e Abastecimento. Portaria n° 210 de 10 de novembro de 1998. Aprova o Regulamento Técnico da Inspeção Tecnológica e Higiênico-Sanitária de Carne de Aves 1998.

[3] Muchon JL, Garcia RG, de Gandra ÉRS, de Assunção ASA, Komiyama CM, Caldara FR (2019). Origin of broiler carcass condemnations. Rev Bras Zootec.

[4] Souza APO, Taconeli CA, Plugge NF, Molento CFM (2018). Broiler chicken meat inspection data in Brazil: a first glimpse into an animal welfare approach. Rev Bras Cienc Avic.

[5] Souza MCC, Borges LFNM, Nascimento YF, Costa LRM, Dias SC, Ventura NKO (2021). Time series evaluation of ascitic syndrome condemnation at poultry abattoirs under Federal Inspection Service of Brazil (2010-2019). Pesqui Veterinária Bras.

[6] Buzdugan SN, Chang YM, Huntington B, Rushton J, Guitian J, Alarcon P (2020). Identification of production chain risk factors for slaughterhouse condemnation of broiler chickens. Prev Vet Med.

[7] Jaguezeski AM, Engelmann AM, Machado INDR, Batti BPB (2020). The effect of four commercial broiler hybrids and the season on occurrence of broiler condemnations in the abattoirs. Cienc Rural.

[8] dos Santos Candido MJ, Zanini SF, de Ferreira MF, de Araujo FAC, Moreira Teixeira AP, Cipriano RC (2021). Main causes of chicken carcass condemnations in Espírito Santo, Brazil. Semin Agrar.

[9] Bernd KS, Kump AW-S, Freise F, Reich F, Kehrenberg C (2003). Influences of biosecurity on the occurrence of cellulitis in broiler flocks. J Appl Poult Res.

[10] Saraiva S, Saraiva C, Oliveira I, Stilwell G, Esteves A (2021). Effects of age, weight, and housing system on prevalence of dead on arrival and carcass condemnation causes in laying hens. Poult Sci [Internet].

[11] Schulze Bernd K, Wilms-Schulze Kump A, Rohn K, Reich F, Kehrenberg C (2020). Management factors influencing the occurrence of cellulitis in broiler chickens. Prev Vet Med.

[12] Belintani R, Garcia RG, de Alencar NI, Borille R, Sgavioli S, Caldara FR (2019). Broiler carcass condemnation pattern during processing. Rev Bras Zootec.

[13] Ahmed MAB, Abdelgadir AE, Ismail HM (2021). Estimation of Knowledge , Attitude , and Practice Related ( KAP ) to Biosecurity Measures and Hazard Analysis Critical Control Point ( HACCP ) Prerequisites in Poultry Meat Production in Khartoum State , Sudan. J Anim Sci Livest Prod.

[14] Descovich K, Li X, Sinclair M, Wang Y, Phillips CJC (2019). The effect of animal welfare training on the knowledge and attitudes of abattoir stakeholders in China. Animals..

[15] Drohomeretski E, Gouvea Da Costa SE, Pinheiro De Lima E, Garbuio PADR (2014). Lean, six sigma and lean six sigma: an analysis based on operations strategy. Int J Prod Res.

[16] Arruda JNT, Mendes AS, Guirro ECBP, Schneider M, Sikorski RR, Sausen L (2016). Live performance, carcass yield, and welfare of broilers of different genetic strains reared at different housing densities. Rev Bras Cienc Avic.

[17] BRASIL. Ministério da Agricultura, Pecuária e Abastecimento. Portaria n^o^ 74, de 7 de maio de 2019. Portaria n^o^ 210, de 10 de novembro de 1998, passa a vigorar com alterações. 2019 p. 1–2.

[18] BRASIL. Ministério da Agricultura, Pecuária e Abastecimento. Decreto no 10.468 de 18 de agosto de 2020. 2020. Available from: https://www.in.gov.br/en/web/dou/-/decreto-n-10.468-de-18-de-agosto-de-2020-272981604

[19] Vecerek V, Vecerkova L, Voslarova E (2019). Comparison of the frequency of patho-anatomic findings in laying hens with findings in broiler chickens and turkeys detected during post-mortem veterinary inspection. Poult Sci.

[20] Garcia DT, Nascimento YF, da Dias SC, Moura AO, Costa PC, do Amaral AB (2021). Microbiological assessment at slaughter of chicken carcasses from commercial, backyard and semi-backyard production systems. J Infect Dev Ctries.

[21] Di Pillo F, Anríquez G, Alarcón P, Jimenez-Bluhm P, Galdames P, Nieto V (2019). Backyard poultry production in Chile: animal health management and contribution to food access in an upper middle-income country. Prev Vet Med.

[22] Večerková L, Voslářová E, Večerek V (2019). Comparison of the welfare of laying hens , broiler chickens and turkeys in terms of bird health as surveyed during inspection in slaughterhouses Meat inspection is one of the most widely implemented and longest running systems of veterinary surveillance. Acta Vet Brno.

[23] De SWF, Granjeiro MDB, Procópio DP (2019). Analysis of the economic loss and the main causes of total condemnation of poultry carcasses under Brazilian federal inspection between 2013 and 2017. Arch Vet Sci.

[24] Marchewka J, Vasdal G, Moe RO (2020). Associations between on-farm welfare measures and slaughterhouse data in commercial flocks of Turkey hens (Meleagris gallopavo). Poult Sci.

[25] Iannetti L, Neri D, Santarelli GA, Cotturone G, Podaliri Vulpiani M, Salini R (2020). Animal welfare and microbiological safety of poultry meat: impact of different at-farm animal welfare levels on at-slaughterhouse Campylobacter and Salmonella contamination. Food Control.

[26] Benincasa NC, Sakamoto KS, Silva IJO, Lobos CMV (2020). Animal welfare: impacts of pre-slaughter operations on the current poultry industry. J Anim Behav Biometeorol.

[27] Cockram MS, Dulal KJ (2018). Injury and mortality in broilers during handling and transport to slaughter. Can J Anim Sci.

[28] Grandin T (2010). Welfare during transport of livestock and poultry. Improving animal welfare: a practical approach.

[29] Allain V, Salines M, Le Bouquin S, Magras C (2018). Designing an innovative warning system to support risk-based meat inspection in poultry slaughterhouses. Food Control.

[30] Pilecco M, Almeida Paz ICL, Tabaldi LA, Naas IA, Garcia RG, Caldara FR (2013). Training of catching teams and reduction of Back scratches in broilers. Brazilian J Poult Sci.

[31] BRASIL. Ofício-circular no 104/2020/DIPOA/SDA/MAPA. Poultry. Ante and post-mortem inspection procedures and forms. 2020. 1–11.

[32] Ansari-Lari M, Rezagholi M (2007). Poultry abattoir survey of carcass condemnations in Fars province, southern Iran. Prev Vet Med.

[33] d’Arc Moretti L, Dias RA, Telles EO, Balian S de C. Time series evaluation of traumatic lesions and airsacculitis at one poultry abattoir in the state of São Paulo, Brazil (1996-2005). Prev Vet Med 2010;94(3–4):231–239. Available from: 10.1016/j.prevetmed.2010.02.013. 10.1016/j.prevetmed.2010.02.013 20233633

[34] Baghbanzadeh A, Decuypere E (2008). Ascites syndrome in broilers: physiological and nutritional perspectives. Avian Pathol.

[35] Olkowski AA, Wojnarowicz C, Rathgeber BM, Abbott JA, Classen HL (2003). Lesions of the pericardium and their significance in the aetiology of heart failure in broiler chickens. Res Vet Sci.

[36] Ekstrand C, Carpenter TE (1998). Temporal aspects of foot-pad dermatitis in Swedish broilers.

[37] Salines M, Allain V, Roul H, Magras C, Le Bouquin S (2017). Rates of and reasons for condemnation of poultry carcases: harmonised methodology at the slaughterhouse. Vet Rec.

[38] Garcia RG, Caldara FR, Vargas FM, Graciano JD, Freitas LW, Schwingel AW (2008). Feeding fasting pre-slaughter in yield and quality of carcass of broilers type griller. Rev Agrar.

[39] Ludtke CB, Ciocca JRP, Dandin T, Barbalho PC, Vilela JA (2010). Humane poultry slaughter. WSPA-Soc.

[40] Mukaka MM. Statistics corner: a guide to appropriate use of correlation coefficient in medical research [internet]. Malawi Med J. 2012;24 Available from: www.mmj.medcol.mw. PMC3576830 23638278

